# Assessing the Relationship Between Gut Microbiota and Bone Mineral Density

**DOI:** 10.3389/fgene.2020.00006

**Published:** 2020-01-31

**Authors:** Shiqiang Cheng, Xin Qi, Mei Ma, Lu Zhang, Bolun Cheng, Chujun Liang, Li Liu, Ping Li, Om Prakash Kafle, Yan Wen, Feng Zhang

**Affiliations:** Key Laboratory of Trace Elements and Endemic Diseases of National Health and Family Planning Commission, School of Public Health, Health Science Center, Xi’an Jiaotong University, Xi’an, China

**Keywords:** gut microbiota, bone mineral density, polygenic risk score, osteoporosis, fracture

## Abstract

**Background:**

Recent study demonstrates the comprehensive effects of gut microbiota on complex diseases or traits. However, limited effort has been conducted to explore the potential relationships between gut microbiota and BMD.

**Methods:**

We performed a polygenetic risk scoring (PRS) analysis to systematically explore the relationships between gut microbiota and body BMD. Significant SNP sets associated with gut microbiota were derived from previous genome-wide association study (GWAS). In total, 2,294 to 5,065 individuals with BMD values of different sites and their genotype data were obtained from UK Biobank cohort. The gut microbiota PRS of each individual was computed from the SNP genotype data for each study subject of UK Biobank by PLINK software. Using computed PRS as the instrumental variables of gut microbiota, Pearson correlation analysis of individual PRS values and BMD values was finally conducted to test the potential association between gut microbiota and target trait.

**Results:**

In total, 31 BMD traits were selected as outcome to assess their relationships with gut microbiota. After adjusted for age, sex, body mass index, and the first 5 principal components (PCs) as the covariates using linear regression model, pelvis BMD (*P* = 0.0437) showed suggestive association signal with gut microbiota after multiple testing correction.

**Conclusion:**

Our study findings support the weak relevance of gut microbiota with the development of BMD.

## Introduction

Bone mineral density (BMD) is widely used in clinical practice as an indirect indicator of osteoporosis and fracture risk (Johnell et al., 2005; Kanis et al., 2006). According to a report, there will be more than 2 million osteoporosis-related fractures in the United States in 2005 which costing $17 billion and the annual fractures and costs will increase by nearly 50% in 2025 (Burge et al., 2007). In the developed countries, 2% to 8% of men and 9% to 38% of women are affected by osteoporosis according to the method of diagnosis (Wade et al., 2014).

Evidence from intergenerational studies estimated that genetic factors account for 50–70% of the variance in BMD, whereas twin studies estimated reach 80–90% (Garabedian, 1995; Krall and Dawsonhughes, 2009). A multivariate twin study of Finnish men suggested that the heritability was estimated to account for 75% of femoral BMD variation and 83% of lumbar BMD variation respectively (Videman et al., 2007). Up to now, extensive genetic studies have been conducted to detect genetic factors underlying BMD. For example, genome-wide association studies (GWAS) have identified more than 65 novel genome-wide significant loci for BMD and detected 14 risk loci for fracture (Estrada et al., 2012). In a targeted sequencing of genome-wide significant loci for BMD, *WLS, ARHGAP1*, and 5′ of *MEF2C* were identified much more strongly associated with BMD compared to the GWAS SNPs (Hsu et al., 2016). However, the full genetic mechanism of BMD remains elusive now.

The intestinal microbiota is the complex community of microbes colonizing the gastrointestinal tract. Recently, extensive researches have focused on the human gut microbiota and our knowledge of the resident flora and its potential functional capacity is growing rapidly. It has been reported that human gut microbiota contains tens of trillions of microbes, including at least 1,000 different kinds of known bacteria with more than 3 million genes (Qin et al., 2010). The activity and composition of the gut microbiota co-develop with the host from birth and have complex interactions with the host genome, nutrition, and lifestyle (Nicholson et al., 2012). The changes of composition and abundance of microbiota have been linked with many inflammatory and metabolic disorders, such as inflammatory bowel disease, rheumatoid arthritis, type 2 diabetes, and obesity (Ley et al., 2006; Frank et al., 2007; Qin et al., 2012; Zhang et al., 2015). More recently, Wang et al. conducted a 16S rRNA gene sequencing to detect the composition and diversity changes of gut microbiota in patients with primary osteoporosis and primary osteopenia (Wang et al., 2017). The results suggest that compared with normal controls, the bacterial composition and diversity are altered in osteoporosis and osteopenia patients, which supported the view that the bone health might be influenced by the gut microbiota (Wang et al., 2017). But limited efforts have been conducted to explore the relationship between gut microbiota and BMD of different sites until now.

A polygenic risk score (PRS) is a sum of trait-associated alleles across many genetic loci, typically weighted by effect sizes estimated from a genome-wide association study (Euesden et al., 2014). PRSs are generated by running a GWAS on a discovery sample, selecting SNPs on the basis of their association with the phenotype, and creating a sum of their phenotype-associated alleles (often weighted by the SNP-specific coefficients from the GWAS) that can be evaluated in a separate replication sample (Dudbridge, 2013). Polygenic risk score (PRS) analysis is not only able to evaluate the effects of susceptible loci on disease risks, but also capable of exploring the genetic relationships between various complex diseases and traits (Euesden et al., 2014). The use of PRS has become increasingly popular, facilitating genetic discoveries regarding complex traits. Then, the approach has motivated several other applications, including polygenic Mendelian Randomization (Hung et al., 2014). For instance, PRS analysis has been successfully applied to multiple complex diseases, such as diabetes, sleep traits, and coronary heart disease (Kawai et al., 2017; Carter et al., 2019; Nazarzadeh et al., 2019; Richmond et al., 2019).

In this study, we performed a PRS analysis to systematically explore the relationships between gut microbiota and BMD of different sites. Our results may substantially expand the knowledge of relationships between gut microbiota and the development of BMD.

## Materials and Methods

### UK Biobank Data Set

This study was conducted using the UK Biobank resource. The UK Biobank study is a large prospective cohort study of approximately 500,000 individuals aged between 37 and 76 years (99.5% were aged 40–69 years) from all over of the United Kingdom (Sudlow et al., 2015). All participants provided a range of information on health status, demographics, and lifestyle *via* questionnaires and interviews. UK Biobank has ethical approval from the Northwest Multi-centre Research Ethics Committee, and informed consent was obtained from all participants. Specific for this study, 2,294 to 5,065 individuals with BMD values of 31 different sites were included (**Table 1**). BMD values for all body sites were measured by dual energy X-ray absorptiometry (DXA) (Harvey et al., 2013). All phenotypic values were adjusted for age, sex, body mass index, and the first 5 principal components (PCs) as the covariates using linear regression model. A set of 40 genetic PCs were pre-calculated by the UK Biobank (Bycroft et al., 2018). Briefly, by using a set of 407,219 independent, high-quality samples and 147,604 high-quality markers pruned to minimize linkage disequilibrium, they calculated the corresponding principal component-loadings and projected all samples onto the principal components, thus forming a set of principal component scores for all samples in the cohort (Bycroft et al., 2018). Detailed information regarding the calculation is described elsewhere (Bycroft et al., 2017). The detailed number of samples for these BMD phenotypes can be found in the **Table 1**. The genotypes of the UK Biobank participants were assayed using either the Affymetrix UK BiLEVE Axiom or Affymetrix UK Biobank Axiom array. Imputation was conducted by IMPUTE4 against the reference panel of the Haplotype Reference Consortium, 1000 Genomes, and UK10K projects. We used the imputed genetic data set released by UK Biobank in July 2017 (Bycroft et al., 2018). Full details regarding these data have been published elsewhere (Canela-Xandri et al., 2018). This research has been conducted using the UK Biobank Resource under Application Number 46478. The authors thank all UK Biobank participants and researchers who contributed or collected data.

**Table 1:** The number of samples for the BMD of different sites from the UK Biobank.

Phenotypes	Total samples
Arm BMD (left)	4267
Arm BMD (right)	4267
Arms BMD	5064
Femur lower neck BMD (left)	2295
Femur lower neck BMD (right)	2294
Femur neck BMD (left)	5063
Femur neck BMD (right)	5062
Femur shaft BMD (left)	5046
Femur shaft BMD (right)	5048
Femur total BMD (left)	5046
Femur total BMD (right)	5048
Femur total BMD	5065
Femur troch BMD (left)	5064
Femur troch BMD (right)	5063
Femur upper neck BMD (left)	2298
Femur upper neck BMD (right)	2295
Femur wards BMD (left)	5064
Femur wards BMD (right)	5063
L1-L4 BMD	5065
Leg BMD (left)	4268
Leg BMD (right)	4268
Legs BMD	5065
Pelvis BMD	5065
Ribs BMD	5065
Spine BMD	5065
Total BMD (left)	4268
Total BMD (right)	4268
Total BMD	5065
Trunk BMD (left)	4268
Trunk BMD (right)	4268
Trunk BMD	5065

BMD, bone mineral density.

### GWAS Data Sets of the Gut Microbiota

The GWAS summary data sets of gut microbiota were derived from two previous studies (Goodrich et al., 2016; Turpin et al., 2016). Briefly, 1,561 healthy individuals and 2,731 subjects were recruited in the two GWAS data sets, respectively. The V4 hypervariable region of bacterial 16S rRNA was sequenced in paired-end mode (2 × 150 bp, 2 × 250 bp, respectively) on the Illumina MiSeq platform using primers 515F and 806R. Mate-pair merging, de-multiplexing, quality control, and operational taxonomic units (OTU) picking were performed by QIIME (v1.8.0) pipeline with default parameters (Caporaso et al., 2010). SNP–microbe association was estimated using genome-wide efficient mixed-model association (GEMMA) (Zhou and Stephens, 2012). Specific in our study, 306 significant SNPs at *P <* 5.0 × 10^−5^ were selected for subsequent PRS analysis (**Supplementary Table 1**). Detailed description of experimental design, sample characteristics, genotyping, imputation, and statistical analysis can be found in previous studies (Goodrich et al., 2016; Turpin et al., 2016).

### Polygenic Risk Scores (PRS) Analysis

The significant SNPs with genotype data derived from UK Biobank data set were analyzed in this study. The gut microbiota PRS of each individual was computed from the SNP genotype data for each study individual by PLINK according to the standard approach used by previous studies (Purcell et al., 2007). Let PRS_m_ denotes the PRS value of gut microbiota for the *m*th subject, defined as $PRS_{m}=\sum_{i=1}^{l}\beta_{i}SNP_{im}$. *β_i_* is the effect parameter of risk allele of the *i*th significant SNP associated with gut microbiota, which was obtained from the published study (Goodrich et al., 2016; Turpin et al., 2016). SNP_im_ is the dosage (0, 1, 2) of the risk allele of the *i*th SNP for the *m*th study subject. *l* denotes the total number of gut microbiota analyzed in this study. PLINK 2.0 were used to perform the PRS analysis (http://www.cog-genomics.org/plink/2.0/). Using computed PRS as the instrumental variables of gut microbiota, Pearson correlation analysis of individual PRS values and BMD values was finally used to detect the potential associations between gut microbiota and target traits. *P* < 0.05 was detected to be significant in this study. All statistical analyses were performed using R (https://www.r-project.org/).

## Results

**Figure 1:**
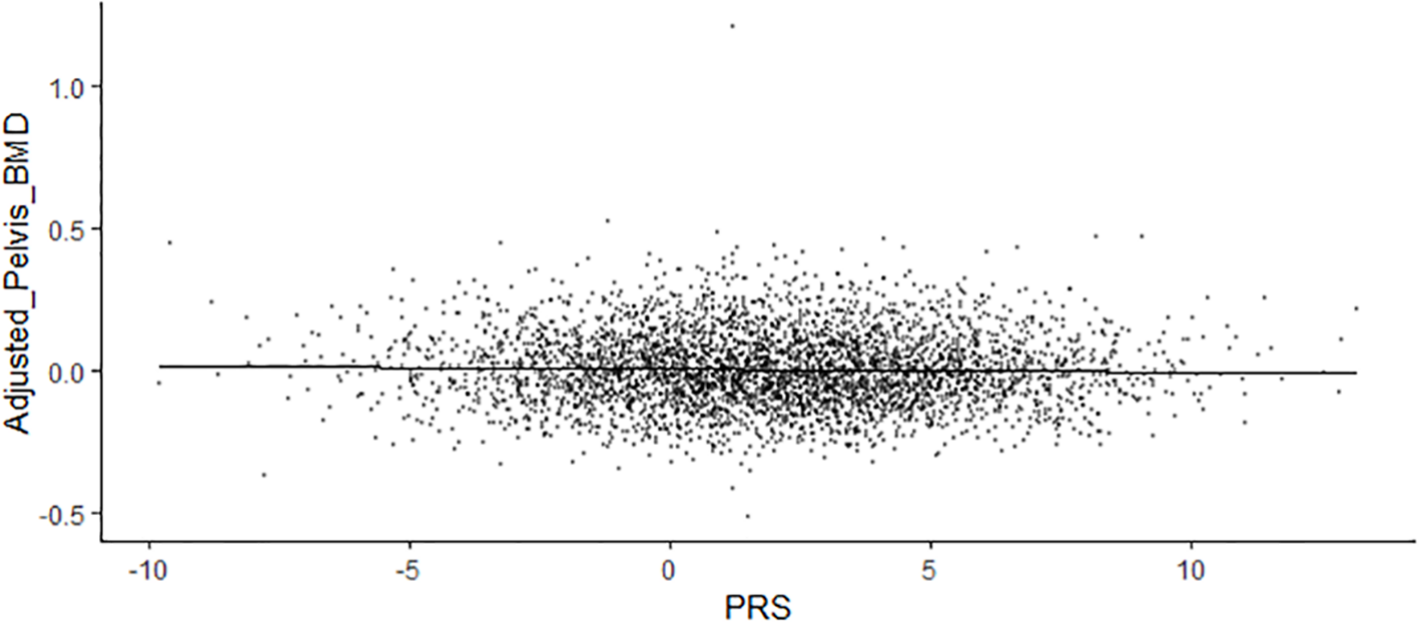
The scatter plot of the adjusted pelvis BMD and gut microbiota PRS.

In total, 31 BMD of different sites were selected as outcome to assess their relationships with gut microbiota. We calculated the gut microbiota PRS of each individual from UK Biobank study. After adjusted for age, sex, body mass index, and the first 5 PCs as the covariates using linear regression model, pelvis BMD (*P* = 0.0437, Pearson correlation coefficients *=* −0.0283) appeared to be associated with gut microbiota (**Table 2**). **Figure 1** shown the scatter plot of the adjusted pelvis BMD and gut microbiota PRS.

**Table 2:** List of the correlation between BMD of 31 different sites and gut microbiota.

Phenotype	Correlation coefficients	*P*
Arm BMD (left)	0.0114	0.4582
Arm BMD (right)	−0.0028	0.8530
Arms BMD	0.0047	0.7389
Femur lower neck BMD (left)	0.0032	0.8765
Femur lower neck BMD (right)	0.0196	0.3487
Femur neck BMD (left)	0.0053	0.7068
Femur neck BMD (right)	0.0108	0.4431
Femur shaft BMD (left)	−0.0017	0.9039
Femur shaft BMD (right)	0.0141	0.3169
Femur total BMD (left)	−0.0055	0.6969
Femur total BMD (right)	0.0105	0.4539
Femur total BMD	−0.0187	0.1842
Femur troch BMD (left)	−0.0103	0.4636
Femur troch BMD (right)	0.0025	0.8604
Femur upper neck BMD (left)	−0.0012	0.9540
Femur upper neck BMD (right)	0.0071	0.7354
Femur wards BMD (left)	−0.0045	0.7516
Femur wards BMD (right)	−0.0014	0.9212
L1-L4 BMD	−0.0202	0.1511
Leg BMD (left)	−0.0107	0.4846
Leg BMD (right)	−0.0070	0.6497
Legs BMD	−0.0053	0.7082
Pelvis BMD	−0.0283	0.0437
Ribs BMD	−0.0043	0.7593
Spine BMD	−0.0086	0.5429
Total BMD (left)	−0.0050	0.7459
Total BMD (right)	−0.0064	0.6755
Total BMD	−0.0045	0.7463
Trunk BMD (left)	−0.0181	0.2374
Trunk BMD (right)	−0.0155	0.3112
Trunk BMD	−0.0164	0.2445

BMD, bone mineral density.

## Discussion

Limited effort has been conducted to explore the potential relationships between gut microbiota and BMD. In this study, we conducted a PRS analysis to systematically explore the relationships between BMD of different sites and gut microbiota. Pelvis BMD appeared to be associated with gut microbiota by the PRS analysis in this study.

The direct evidence that gut microbiota modulates BMD is from a study which compared BMD and microstructure of bone in germ-free versus conventionally raised mice (Sjögren et al., 2012). Seven-week-old germ-free female mice had better femurs bone structure and density than conventionally raised mice: higher trabecular bone volume to tissue volume, and higher trabecular BMD (Sjögren et al., 2012). However, both BMD and cortical cross-sectional area of trabecular decreased when germ-free mice were recolonized with the gut flora which suggesting that the gut flora is a main regulator of bone mass (Sjögren et al., 2012). McCabe et al. (2013) suggested that C57Bl/6J male mice receiving the probiotic Lactobacillus reuteri ATCC PTA 6475 (a candidate probiotic with anti-TNFα activity) for four weeks showed an increase in femoral trabecular BMC, BMD, trabecular spacing, number, and thickness. In addition, bone mineral content (BMC) and BMD in children with a perturbed intestinal microbiota have altered through post-weaning exposure to low-dose penicillin or introduction of low-dose penicillin to their mother in pregnancy (Cox et al., 2014).

Recent studies have revealed that relationships between gut microbiota and osteoporosis. Wang et al. demonstrated that compared with normal controls, the bacterial composition and diversity are altered in osteoporosis and osteopenia patients (Wang et al., 2017). In animal experimentation, bone loss in postmenopausal osteoporosis model is closely related to host immunity, which is affected by the gut microbiota (Li et al., 2016). The effects of the gut microbiota on bone metabolism provide a promising target for the management of postmenopausal osteoporosis (Li et al., 2016).

Gut microbiota can affect bone metabolism, but its exact mechanism remains unclear now. So far, there are three hypotheses for the mechanisms by which gut microbes regulate bone metabolism, including effects on the immune system, the endocrine system, and calcium absorption. (1) The intestinal microbiota regulates bone metabolism through the immune system. For example, segmental filamentous bacteria in the mouse gut promote the production of IL-17 and IFN-γ, which plays a vital role in the formation of osteoclasts and osteoblasts (Adamopoulos et al., 2010; Duque et al., 2011). (2) The gut microbiota regulates bone metabolism through the endocrine system. In animal experiments, intestinal microbial colonization in sterile mice significantly increased the levels of serum IGF-1 which leading to normalized bone mass and bone growth (Yan et al., 2016). (3) The gut microbiota regulates bone metabolism by affecting the absorption of calcium. In the Caco-2 cell culture model, a special probiotic, such as Lactobacillus saliva, stimulates intestinal cells to absorb calcium (Gilman and Cashman, 2006). Ultimately, it leads to reduced osteoclast activity and/or increased osteoblast activity, which results in increased bone structure, density, and strength (McCabe et al., 2015).

This is the first systematic study of the relationship between gut microbiota and BMD of different sites. However, our study does have certain limitations. Firstly, the gut microbiota related SNP sets were obtained from previous published GWAS. Due to the very limited GWAS of gut microbiota performed at different platforms, some loci which regulate the gut microbiota have not been found until now, which may affect the accuracy of our results. So more GWAS of gut microbiota are needed to illustrate the interactions between gut microbiota and host genetics. Second, all subjects in this study are from European ancestry. Therefore, it should be careful to extrapolate our study findings to other ethnic groups. Third, it will be helpful to understand the relationship of BMD and gut microbiota if the correlation between the PRS and gut microbiota in two previous GAW studies can be calculated. However, we are not permitted to obtain the raw data of the individual level GWAS data of gut microbiota.

In summary, we systematically evaluated the associations between gut microbiota and BMD of different sites utilizing UK Biobank individual level BMD and genotype data of BMD and publicly available GWAS summary data of gut microbiota. We observed modest associations between gut microbiota and pelvis BMD, supporting the weak relevance of gut microbiota with the development of BMD. Our results may help to uncover the roles of gut microbiota on the development of BMD.

## Data Availability Statement

The datasets analyzed in this article are not publicly available. Requests to access the datasets should be directed to fzhxjtu@mail.xjtu.edu.cn.

## Ethics Statement

There is no ethical statement here, because of all data downloaded from the Internet.

## Author Contributions

SC and FZ conceived and designed the study, and wrote the manuscript. SC and FZ collected the data and carried out the statistical analyses. YW, MM, LZ, BC, XQ, CL, LL, PL, and OK made preparations for the manuscript at first. All authors reviewed and approved the final manuscript.

## Funding

This study is supported by the National Natural Scientific Foundation of China (81673112), the Key projects of international cooperation among governments in scientific and technological innovation (2016YFE0119100), the Natural Science Basic Research Plan in Shaanxi Province of China (2017JZ024), and the Fundamental Research Funds for the Central Universities.

## Conflict of Interest

The authors declare that the research was conducted in the absence of any commercial or financial relationships that could be construed as a potential conflict of interest.
