# Supplementary material for: Assessing the Relationship Between Gut Microbiota and Bone Mineral Density
Source: Front Genet. 2020 Jan 31;11:6. doi: 10.3389/fgene.2020.00006 (PMC7005253; doi:10.3389/fgene.2020.00006)
Supplement: Supplementary file 1 [file Table_1.docx]

**Table S1.** The significant SNPs associated with gut microbiota

| **Bacteria** | **CHR** | **SNP** | **A1** | **A2** | **MAF** | **BETA** | **SE** |
| --- | --- | --- | --- | --- | --- | --- | --- |
| k__Bacteria.p__Actinobacteria.c__Coriobacteriia.o__Coriobacteriales.f__Coriobacteriaceae.g__Eggerthella | 1 | rs1376236 | C | A | 0.085 | 16.30 | 2.87 |
| k__Bacteria; p__Bacteroidetes; c__Bacteroidia; o__Bacteroidales; f__Rikenellaceae; g__; s__ | 1 | rs17098734 | G | A | 0.11 | 2.04 | 0.40 |
| k__Bacteria; p__Bacteroidetes; c__Bacteroidia; o__Bacteroidales; f__Porphyromonadaceae; g__Parabacteroides; s__ | 1 | rs10493180 | T | A | 0.161 | -0.23 | 0.04 |
| k__Bacteria; p__Firmicutes; c__Clostridia; o__Clostridiales; f__Ruminococcaceae; g__; s__ | 1 | rs232905 | G | A | 0.104 | 4.49 | 0.88 |
| k__Bacteria; p__Actinobacteria; c__Coriobacteriia; o__Coriobacteriales; f__Coriobacteriaceae; g__Eggerthella; s__lenta | 1 | rs1376235 | G | C | 0.083 | 21.10 | 3.81 |
| k__Bacteria.p__Firmicutes.c__Bacilli | 1 | rs4342835 | A | C | 0.32 | -0.37 | 0.07 |
| k__Bacteria; p__Firmicutes; c__Clostridia; o__Clostridiales; f__; g__; s__ | 1 | rs7524581 | T | C | 0.351 | -14.70 | 2.75 |
| k__Bacteria; p__Firmicutes; c__Clostridia; o__Clostridiales; f__Lachnospiraceae; g__Coprococcus; s__ | 1 | rs6656158 | T | C | 0.124 | 0.31 | 0.06 |
| k__Bacteria; p__Firmicutes; c__Clostridia; o__Clostridiales; f__; g__; s__ | 1 | rs7527642 | A | C | 0.058 | -19.90 | 3.76 |
| k__Bacteria; p__Firmicutes; c__Clostridia; o__Clostridiales; f__Ruminococcaceae; g__; s__ | 1 | rs4620568 | G | C | 0.334 | -0.11 | 0.02 |
| k__Bacteria; p__Firmicutes; c__Clostridia; o__Clostridiales; f__Lachnospiraceae; g__; s__ | 1 | rs12407389 | G | C | 0.382 | -0.95 | 0.20 |
| k__Bacteria; p__Firmicutes; c__Clostridia; o__Clostridiales; f__[Mogibacteriaceae]; g__; s__ | 1 | rs10915971 | T | C | 0.334 | 0.65 | 0.14 |
| k__Bacteria; p__Bacteroidetes; c__Bacteroidia; o__Bacteroidales; f__Bacteroidaceae; g__Bacteroides; s__ | 1 | rs10801316 | A | G | 0.214 | -4.65 | 0.92 |
| k__Bacteria; p__Bacteroidetes; c__Bacteroidia; o__Bacteroidales; f__Bacteroidaceae; g__Bacteroides; s__ | 1 | rs7886938 | A | G | 0.168 | 0.39 | 0.08 |
| k__Bacteria; p__Firmicutes; c__Clostridia; o__Clostridiales; f__Ruminococcaceae; g__; s__ | 1 | rs687746 | A | G | 0.154 | 1.57 | 0.32 |
| k__Bacteria; p__Bacteroidetes; c__Bacteroidia; o__Bacteroidales; f__Bacteroidaceae; g__Bacteroides | 1 | rs737301 | A | G | 0.35 | -3.75 | 0.78 |
| k__Bacteria; p__Firmicutes; c__Clostridia; o__Clostridiales; f__Lachnospiraceae | 1 | rs6674509 | A | G | 0.122 | -0.33 | 0.07 |
| k__Bacteria.p__Firmicutes.c__Clostridia.o__Clostridiales.f__Lachnospiraceae.g__Roseburia | 1 | rs12128018 | C | T | 0.132 | 0.15 | 0.03 |
| k__Bacteria; p__Firmicutes; c__Clostridia; o__Clostridiales; f__Lachnospiraceae; g__; s__ | 1 | rs4565719 | C | T | 0.377 | -1.59 | 0.34 |
| k__Bacteria; p__Firmicutes; c__Clostridia; o__Clostridiales; f__; g__; s__ | 1 | rs185902 | C | T | 0.423 | 11.70 | 2.66 |
| k__Bacteria_p__Firmicutes_c__Clostridia_o__Clostridiales_f__Peptococcaceae_Other | 1 | rs143179968 | T |  | 0.07 | -0.23 | 0.18 |
| k__Bacteria_p__Firmicutes_c__Clostridia_o__Clostridiales_f__Veillonellaceae_g__Megamonas | 1 | rs6683259 | A |  | 0.05 | -0.10 | 0.20 |
| k__Bacteria.p__Actinobacteria.c__Coriobacteriia.o__Coriobacteriales.f__Coriobacteriaceae.g__Slackia | 1 | rs6692744 | C |  | 0.10 | -0.03 | 0.18 |
| k__Bacteria_p__Firmicutes_c__Bacilli_o__Lactobacillales_f__Lactobacillaceae_g__Lactobacillus | 1 | chr1:60346014:D | G |  | 0.05 | 0.06 | 0.21 |
| k__Archaea_p__Euryarchaeota_c__Methanobacteria_o__Methanobacteriales_f__Methanobacteriaceae_g__Methanobrevibacter | 1 | rs6666120 | G |  | 0.31 | 0.07 | 0.10 |
| k__Bacteria.p__Firmicutes.c__Clostridia.o__Clostridiales.f__Veillonellaceae.g__Acidaminococcus* | 1 | rs41269155 | A |  | 0.08 | 0.16 | 0.19 |
| k__Bacteria.p__Firmicutes.c__Clostridia.o__Clostridiales.f__.Mogibacteriaceae..g__Mogibacterium | 1 | rs55987656 | C |  | 0.06 | 0.55 | 0.18 |
| k__Bacteria.p__Actinobacteria.c__Actinobacteria | 2 | rs1446585 | G | A | 0.265 | 0.19 | 0.03 |
| k__Bacteria; p__Firmicutes; c__Clostridia; o__Clostridiales; f__; g__; s__ | 2 | rs7587067 | T | C | 0.317 | -0.10 | 0.02 |
| k__Bacteria; p__Bacteroidetes; c__Bacteroidia; o__Bacteroidales; f__Bacteroidaceae; g__Bacteroides; s__ | 2 | rs10928081 | T | C | 0.135 | 5.57 | 1.11 |
| k__Bacteria; p__Firmicutes; c__Clostridia; o__Clostridiales; f__Ruminococcaceae; g__; s__ | 2 | rs17369648 | T | C | 0.216 | -0.86 | 0.17 |
| k__Bacteria; p__Firmicutes; c__Clostridia; o__Clostridiales; f__Ruminococcaceae; g__; s__ | 2 | rs898031 | T | C | 0.338 | 1.11 | 0.24 |
| k__Bacteria; p__Firmicutes; c__Clostridia; o__Clostridiales; f__Lachnospiraceae | 2 | rs7602355 | T | C | 0.212 | 0.23 | 0.06 |
| k__Bacteria; p__Firmicutes; c__Clostridia; o__Clostridiales; f__Lachnospiraceae; g__; s__ | 2 | rs11125721 | A | G | 0.39 | -0.40 | 0.08 |
| k__Bacteria; p__Firmicutes; c__Clostridia; o__Clostridiales; f__; g__; s__ | 2 | rs10221827 | C | G | 0.372 | -1.47 | 0.29 |
| k__Bacteria; p__Firmicutes; c__Clostridia; o__Clostridiales; f__Lachnospiraceae; g__Blautia; s__ | 2 | rs4673912 | T | G | 0.389 | 1.23 | 0.25 |
| k__Bacteria; p__Firmicutes; c__Clostridia; o__Clostridiales; f__Ruminococcaceae; g__; s__ | 2 | rs7566589 | A | G | 0.419 | -0.30 | 0.06 |
| k__Bacteria; p__Firmicutes; c__Clostridia; o__Clostridiales; f__Lachnospiraceae | 2 | rs11126024 | A | G | 0.19 | 0.28 | 0.06 |
| k__Bacteria; p__Firmicutes; c__Clostridia; o__Clostridiales; f__Lachnospiraceae; g__; s__ | 2 | rs7604350 | A | G | 0.184 | 0.32 | 0.07 |
| k__Bacteria; p__Firmicutes; c__Clostridia; o__Clostridiales; f__; g__; s__ | 2 | rs6542797 | C | T | 0.376 | 0.21 | 0.04 |
| k__Bacteria; p__Firmicutes; c__Clostridia; o__Clostridiales; f__Ruminococcaceae; g__; s__ | 2 | rs2278067 | G | T | 0.487 | 0.24 | 0.04 |
| k__Bacteria; p__Bacteroidetes; c__Bacteroidia; o__Bacteroidales; f__Bacteroidaceae; g__Bacteroides; s__ | 2 | rs2704557 | G | T | 0.483 | -0.60 | 0.12 |
| k__Bacteria; p__Firmicutes; c__Clostridia; o__Clostridiales; f__Ruminococcaceae; g__; s__ | 2 | rs1221172 | C | T | 0.48 | -0.31 | 0.06 |
| k__Bacteria; p__Firmicutes; c__Clostridia; o__Clostridiales; f__Lachnospiraceae; g__Coprococcus; s__ | 2 | rs786402 | C | T | 0.068 | -0.49 | 0.10 |
| k__Bacteria; p__Firmicutes; c__Clostridia; o__Clostridiales; f__Lachnospiraceae | 2 | rs4848184 | C | T | 0.213 | -0.27 | 0.06 |
| k__Bacteria.p__Firmicutes.c__Clostridia.o__Clostridiales.f__Ruminococcaceae | 2 | rs4663855 | G | T | 0.185 | 0.03 | 0.01 |
| k__Bacteria.p__Proteobacteria.c__Betaproteobacteria.o__Burkholderiales | 2 | rs2925216 | T |  | 0.06 | -0.36 | 0.75 |
| k__Bacteria.p__Actinobacteria.c__Coriobacteriia.o__Coriobacteriales.f__Coriobacteriaceae.g__Slackia | 2 | rs10202734 | T |  | 0.07 | 0.02 | 0.21 |
| k__Bacteria.p__Lentisphaerae | 2 | rs111870934 | A |  | 0.10 | 0.03 | 0.19 |
| k__Bacteria; p__Firmicutes; c__Clostridia; o__Clostridiales; f__Ruminococcaceae | 3 | rs12489943 | G | A | 0.228 | 2.75 | 0.51 |
| k__Bacteria; p__Bacteroidetes; c__Bacteroidia; o__Bacteroidales; f__Bacteroidaceae; g__Bacteroides; s__ | 3 | rs360402 | G | A | 0.321 | 0.42 | 0.09 |
| k__Bacteria; p__Firmicutes; c__Clostridia; o__Clostridiales; f__[Mogibacteriaceae]; g__; s__ | 3 | rs13100072 | C | A | 0.293 | 0.71 | 0.15 |
| k__Bacteria; p__Proteobacteria; c__Gammaproteobacteria; o__Pasteurellales; f__Pasteurellaceae; g__Haemophilus | 3 | rs938421 | G | A | 0.126 | -1.50 | 0.32 |
| k__Bacteria; p__Firmicutes; c__Clostridia; o__Clostridiales; f__Lachnospiraceae; g__Lachnospira; s__ | 3 | rs493456 | G | C | 0.277 | 1.24 | 0.23 |
| k__Bacteria; p__Bacteroidetes; c__Bacteroidia; o__Bacteroidales; f__Bacteroidaceae; g__Bacteroides; s__ | 3 | rs13067391 | T | C | 0.088 | -3.64 | 0.69 |
| k__Bacteria; p__Firmicutes; c__Clostridia; o__Clostridiales; f__Lachnospiraceae; g__; s__ | 3 | rs1523451 | T | C | 0.11 | 0.59 | 0.11 |
| k__Bacteria; p__Bacteroidetes; c__Bacteroidia; o__Bacteroidales; f__Bacteroidaceae; g__Bacteroides; s__ | 3 | rs1489848 | A | C | 0.314 | -38.40 | 7.64 |
| k__Bacteria; p__Firmicutes; c__Clostridia; o__Clostridiales; f__Lachnospiraceae; g__; s__ | 3 | rs6772506 | T | C | 0.058 | -0.78 | 0.17 |
| k__Bacteria; p__Firmicutes; c__Clostridia; o__Clostridiales; f__; g__; s__ | 3 | rs16845116 | T | C | 0.141 | 16.20 | 3.82 |
| k__Bacteria; p__Firmicutes; c__Clostridia; o__Clostridiales; f__Ruminococcaceae; g__Faecalibacterium; s__prausnitzii | 3 | rs2136233 | T | G | 0.1 | 0.96 | 0.18 |
| k__Bacteria.p__Firmicutes.c__Clostridia.o__Clostridiales.f__Clostridiaceae | 3 | rs11710273 | A | G | 0.063 | -0.22 | 0.04 |
| k__Bacteria; p__Firmicutes; c__Clostridia; o__Clostridiales; f__Ruminococcaceae; g__; s__ | 3 | rs7646523 | C | T | 0.345 | 1.10 | 0.21 |
| k__Bacteria.p__Firmicutes.c__Clostridia.o__Clostridiales.f__Clostridiaceae | 3 | rs1405597 | G | T | 0.446 | 0.11 | 0.02 |
| k__Bacteria; p__Bacteroidetes; c__Bacteroidia; o__Bacteroidales; f__Bacteroidaceae; g__Bacteroides; s__ | 3 | rs7634041 | C | T | 0.208 | -3.42 | 0.71 |
| k__Bacteria; p__Firmicutes; c__Clostridia; o__Clostridiales; f__Ruminococcaceae; g__; s__ | 3 | rs9790095 | G | T | 0.084 | -0.21 | 0.05 |
| k__Bacteria_p__Firmicutes_c__Clostridia_o__Clostridiales_f__Lachnospiraceae_g__Pseudobutyrivibrio | 3 | rs13100905 | T |  | 0.05 | -0.27 | 0.21 |
| k__Bacteria.p__Firmicutes.c__Clostridia.o__Clostridiales.f__Veillonellaceae.g__Acidaminococcus | 3 | rs62273067 | G |  | 0.39 | 0.01 | 0.10 |
| k__Bacteria_p__Firmicutes_c__Clostridia_o__Clostridiales_f__Veillonellaceae_g__Megamonas | 3 | rs73005672 | T |  | 0.06 | 0.12 | 0.19 |
| k__Bacteria.p__Proteobacteria.c__Gammaproteobacteria.o__Pasteurellales.f__Pasteurellaceae.g__Aggregatibacter | 3 | rs13081014 | A |  | 0.08 | 0.56 | 0.21 |
| k__Bacteria; p__Bacteroidetes; c__Bacteroidia; o__Bacteroidales; f__Bacteroidaceae; g__Bacteroides; s__ | 4 | rs2866194 | G | A | 0.288 | -0.38 | 0.07 |
| k__Bacteria; p__Firmicutes; c__Erysipelotrichi; o__Erysipelotrichales; f__Erysipelotrichaceae; g__Holdemania; s__ | 4 | rs3117600 | G | A | 0.266 | -0.11 | 0.02 |
| k__Bacteria; p__Bacteroidetes; c__Bacteroidia; o__Bacteroidales; f__Bacteroidaceae; g__Bacteroides | 4 | rs6536782 | G | A | 0.208 | -4.18 | 0.96 |
| k__Bacteria; p__Firmicutes; c__Clostridia; o__Clostridiales; f__; g__; s__ | 4 | rs1125819 | T | C | 0.216 | 13.60 | 3.08 |
| k__Bacteria; p__Firmicutes; c__Clostridia; o__Clostridiales; f__Lachnospiraceae; g__; s__ | 4 | rs987694 | A | G | 0.419 | -2.67 | 0.51 |
| k__Bacteria; p__Firmicutes; c__Clostridia; o__Clostridiales; f__Lachnospiraceae; g__; s__ | 4 | rs3892490 | A | G | 0.21 | 0.48 | 0.10 |
| k__Bacteria; p__Firmicutes; c__Clostridia; o__Clostridiales; f__; g__; s__ | 4 | rs4699323 | A | G | 0.276 | -13.50 | 3.03 |
| k__Bacteria; p__Firmicutes; c__Clostridia; o__Clostridiales; f__Lachnospiraceae; g__; s__ | 4 | rs10517198 | A | G | 0.126 | -0.38 | 0.09 |
| k__Bacteria; p__Firmicutes; c__Clostridia; o__Clostridiales; f__Ruminococcaceae; g__Faecalibacterium; s__prausnitzii | 4 | rs1346183 | C | T | 0.105 | -0.10 | 0.02 |
| k__Bacteria; p__Bacteroidetes; c__Bacteroidia; o__Bacteroidales; f__Bacteroidaceae; g__Bacteroides; s__ | 4 | rs1948223 | C | T | 0.325 | -2.83 | 0.53 |
| k__Bacteria; p__Firmicutes; c__Clostridia; o__Clostridiales; f__; g__; s__ | 4 | rs11733214 | C | T | 0.286 | -15.60 | 2.93 |
| k__Bacteria; p__Bacteroidetes; c__Bacteroidia; o__Bacteroidales; f__Bacteroidaceae; g__Bacteroides; s__ | 4 | rs17506987 | G | T | 0.151 | -4.00 | 0.80 |
| k__Bacteria.p__Firmicutes.c__Bacilli.o__Lactobacillales.f__Leuconostocaceae.g__Weissella | 4 | rs3775467 | A |  | 0.07 | -0.06 | 0.20 |
| k__Bacteria.p__Proteobacteria.c__Gammaproteobacteria.o__Pseudomonadales.f__Moraxellaceae.g__Acinetobacter | 4 | rs6534322 | A |  | 0.06 | 0.02 | 0.19 |
| k__Bacteria; p__Firmicutes; c__Clostridia; o__Clostridiales; f__Ruminococcaceae; g__; s__ | 5 | rs7732898 | C | A | 0.207 | -0.84 | 0.16 |
| k__Bacteria.p__Proteobacteria.c__Gammaproteobacteria.o__Pasteurellales | 5 | rs2166343 | G | A | 0.379 | 1.07 | 0.20 |
| k__Bacteria; p__Firmicutes; c__Clostridia; o__Clostridiales; f__Lachnospiraceae; g__; s__ | 5 | rs1684851 | C | A | 0.279 | -0.39 | 0.09 |
| k__Bacteria; p__Bacteroidetes; c__Bacteroidia; o__Bacteroidales; f__Bacteroidaceae; g__Bacteroides; s__ | 5 | rs295569 | T | C | 0.343 | -0.40 | 0.09 |
| k__Bacteria; p__Bacteroidetes; c__Bacteroidia; o__Bacteroidales; f__Rikenellaceae; g__; s__ | 5 | rs3909540 | T | G | 0.098 | -1.13 | 0.21 |
| k__Bacteria; p__Firmicutes; c__Clostridia; o__Clostridiales; f__Ruminococcaceae; g__; s__ | 5 | rs3733890 | A | G | 0.301 | -0.05 | 0.01 |
| k__Bacteria; p__Firmicutes; c__Clostridia; o__Clostridiales; f__Lachnospiraceae; g__; s__ | 5 | rs17145135 | A | G | 0.056 | -0.77 | 0.17 |
| k__Bacteria; p__Firmicutes; c__Clostridia; o__Clostridiales; f__Ruminococcaceae; g__; s__ | 5 | rs2447859 | A | G | 0.198 | -2.98 | 0.66 |
| k__Bacteria; p__Bacteroidetes; c__Bacteroidia; o__Bacteroidales; f__Bacteroidaceae; g__Bacteroides; s__ | 5 | rs2228440 | A | G | 0.102 | 0.61 | 0.14 |
| k__Bacteria.p__Firmicutes.c__Clostridia.o__Clostridiales.f__Clostridiaceae.g__ | 5 | rs10055309 | C | T | 0.059 | 0.35 | 0.06 |
| k__Bacteria; p__Bacteroidetes; c__Bacteroidia; o__Bacteroidales; f__Bacteroidaceae; g__Bacteroides; s__ | 5 | rs159373 | G | T | 0.057 | 8.35 | 1.59 |
| k__Bacteria_p__Firmicutes_c__Clostridia_o__Clostridiales_f__Lachnospiraceae_g__Pseudobutyrivibrio | 5 | rs1826812 | T |  | 0.05 | -0.28 | 0.20 |
| k__Bacteria_p__Bacteroidetes_c__Bacteroidia_o__Bacteroidales_f__.Barnesiellaceae* | 5 | rs16901246 | T |  | 0.11 | -0.13 | 0.18 |
| k__Bacteria.p__Actinobacteria.c__Actinobacteria.o__Actinomycetales.f__Micrococcaceae | 5 | rs113045860 | G |  | 0.06 | 0.02 | 0.22 |
| k__Bacteria_p__Firmicutes_c__Clostridia_o__SHA.98 | 5 | rs34869836 | A |  | 0.32 | 0.03 | 0.10 |
| k__Bacteria.p__Firmicutes.c__Clostridia.o__Clostridiales.f__Veillonellaceae.g__Acidaminococcus | 5 | chr5:90301196:I | TA |  | 0.05 | 0.45 | 0.24 |
| k__Bacteria; p__Firmicutes; c__Clostridia; o__Clostridiales; f__Lachnospiraceae; g__; s__ | 6 | rs1889879 | C | A | 0.37 | 0.40 | 0.08 |
| k__Bacteria.p__Firmicutes.c__Clostridia.o__Clostridiales.f__Lachnospiraceae.g__Roseburia | 6 | rs9397979 | G | A | 0.243 | -0.12 | 0.03 |
| k__Bacteria; p__Firmicutes; c__Clostridia; o__Clostridiales; f__; g__; s__ | 6 | rs9321334 | G | A | 0.274 | 13.70 | 2.99 |
| k__Bacteria.p__Proteobacteria.c__Gammaproteobacteria.o__Pasteurellales.f__Pasteurellaceae.g__Haemophilus | 6 | rs16880682 | T | A | 0.054 | 2.05 | 0.45 |
| k__Bacteria; p__Firmicutes; c__Clostridia; o__Clostridiales; f__Lachnospiraceae; g__; s__ | 6 | rs6901947 | G | A | 0.339 | 0.37 | 0.08 |
| k__Bacteria; p__Firmicutes; c__Clostridia; o__Clostridiales; f__Lachnospiraceae; g__; s__ | 6 | rs7744601 | G | A | 0.187 | -0.31 | 0.07 |
| k__Bacteria; p__Firmicutes; c__Clostridia; o__Clostridiales; f__Lachnospiraceae | 6 | rs9402233 | G | A | 0.157 | 0.26 | 0.06 |
| k__Bacteria; p__Proteobacteria; c__Gammaproteobacteria; o__Enterobacteriales; f__Enterobacteriaceae; g__; s__ | 6 | rs7749517 | T | C | 0.134 | 12.50 | 2.33 |
| k__Bacteria; p__Firmicutes; c__Clostridia; o__Clostridiales; f__; g__; s__ | 6 | rs9397927 | G | C | 0.261 | 3.63 | 0.68 |
| k__Bacteria; p__Firmicutes; c__Clostridia; o__Clostridiales; f__Ruminococcaceae; g__; s__ | 6 | rs7761311 | T | C | 0.384 | -3.01 | 0.58 |
| k__Bacteria; p__Firmicutes; c__Clostridia; o__Clostridiales; f__Ruminococcaceae | 6 | rs9397756 | T | C | 0.307 | -2.37 | 0.46 |
| k__Bacteria; p__Firmicutes; c__Clostridia; o__Clostridiales; f__Lachnospiraceae; g__; s__ | 6 | rs9373001 | T | C | 0.216 | 2.09 | 0.41 |
| k__Bacteria; p__Bacteroidetes; c__Bacteroidia; o__Bacteroidales; f__Bacteroidaceae; g__Bacteroides; s__ | 6 | rs380915 | T | C | 0.219 | 2.10 | 0.43 |
| k__Bacteria; p__Firmicutes; c__Clostridia; o__Clostridiales; f__; g__; s__ | 6 | rs2269706 | A | G | 0.067 | 2.09 | 0.38 |
| k__Bacteria; p__Firmicutes; c__Clostridia; o__Clostridiales; f__Lachnospiraceae; g__; s__ | 6 | rs6914260 | A | G | 0.178 | -0.53 | 0.10 |
| k__Bacteria; p__Proteobacteria; c__Gammaproteobacteria; o__Enterobacteriales; f__Enterobacteriaceae; g__; s__ | 6 | rs13214269 | C | G | 0.134 | 8.72 | 1.70 |
| k__Bacteria; p__Firmicutes; c__Clostridia; o__Clostridiales; f__; g__; s__ | 6 | rs10484857 | A | G | 0.076 | 15.10 | 3.04 |
| k__Bacteria.p__Firmicutes.c__Clostridia.o__Clostridiales.f__Ruminococcaceae | 6 | rs1204250 | A | G | 0.396 | 0.03 | 0.01 |
| k__Bacteria; p__Verrucomicrobia; c__Verrucomicrobiae; o__Verrucomicrobiales; f__Verrucomicrobiaceae; g__Akkermansia; s__muciniphila | 6 | rs10081087 | A | G | 0.26 | 0.57 | 0.12 |
| k__Bacteria; p__Firmicutes; c__Clostridia; o__Clostridiales; f__[Mogibacteriaceae]; g__; s__ | 6 | rs6933878 | C | G | 0.224 | -0.74 | 0.16 |
| k__Bacteria; p__Firmicutes; c__Clostridia; o__Clostridiales; f__Ruminococcaceae; g__; s__ | 6 | rs11969591 | C | T | 0.062 | -2.06 | 0.41 |
| k__Bacteria; p__Firmicutes; c__Clostridia; o__Clostridiales; f__Lachnospiraceae; g__Blautia; s__ | 6 | rs6929224 | C | T | 0.146 | 0.25 | 0.05 |
| k__Bacteria; p__Bacteroidetes; c__Bacteroidia; o__Bacteroidales; f__Bacteroidaceae; g__Bacteroides; s__ | 6 | rs2198863 | A | T | 0.177 | -0.54 | 0.11 |
| k__Bacteria; p__Firmicutes; c__Clostridia; o__Clostridiales; f__; g__; s__ | 6 | rs9350764 | G | T | 0.333 | -0.13 | 0.03 |
| k__Bacteria; p__Firmicutes; c__Clostridia; o__Clostridiales; f__Ruminococcaceae; g__; s__ | 6 | rs4548008 | G | T | 0.208 | 0.30 | 0.06 |
| k__Bacteria; p__Firmicutes; c__Clostridia; o__Clostridiales; f__; g__; s__ | 6 | rs6933411 | C | T | 0.336 | 11.80 | 2.75 |
| k__Bacteria_p__Firmicutes_c__Bacilli_o__Lactobacillales_f__Lactobacillaceae_g__Lactobacillus | 6 | chr6:80338468:I | GA |  | 0.06 | 0.11 | 0.17 |
| k__Bacteria.p__Proteobacteria.c__Gammaproteobacteria.o__Pasteurellales.f__Pasteurellaceae.g__Aggregatibacter | 6 | chr6:23943584:I | T |  | 0.09 | 0.22 | 0.18 |
| k__Bacteria; p__Bacteroidetes; c__Bacteroidia; o__Bacteroidales; f__Bacteroidaceae; g__Bacteroides; s__ | 7 | rs4628206 | T | A | 0.143 | -55.40 | 10.10 |
| k__Bacteria; p__Firmicutes; c__Clostridia; o__Clostridiales; f__Lachnospiraceae; g__; s__ | 7 | rs10229688 | G | A | 0.064 | -3.15 | 0.58 |
| k__Bacteria; p__Firmicutes; c__Clostridia; o__Clostridiales; f__; g__; s__ | 7 | rs17170765 | G | A | 0.109 | 4.07 | 0.87 |
| k__Bacteria; p__Firmicutes; c__Clostridia; o__Clostridiales; f__Lachnospiraceae | 7 | rs13223655 | G | A | 0.248 | 0.25 | 0.05 |
| k__Bacteria; p__Firmicutes; c__Clostridia; o__Clostridiales; f__Ruminococcaceae; g__; s__ | 7 | rs206196 | T | C | 0.42 | -0.05 | 0.01 |
| k__Bacteria; p__Firmicutes; c__Clostridia; o__Clostridiales; f__; g__; s__ | 7 | rs4717021 | T | C | 0.358 | 3.42 | 0.65 |
| k__Bacteria.p__Firmicutes.c__Clostridia.o__Clostridiales.f__Lachnospiraceae.g__Roseburia | 7 | rs194522 | T | C | 0.147 | -0.16 | 0.03 |
| k__Bacteria; p__Firmicutes; c__Clostridia; o__Clostridiales; f__Ruminococcaceae; g__; s__ | 7 | rs1636874 | T | C | 0.31 | 1.64 | 0.33 |
| k__Bacteria; p__Firmicutes; c__Clostridia; o__Clostridiales; f__Lachnospiraceae; g__; s__ | 7 | rs12704545 | A | C | 0.476 | 0.92 | 0.19 |
| k__Bacteria; p__Firmicutes; c__Clostridia; o__Clostridiales; f__[Mogibacteriaceae]; g__; s__ | 7 | rs10276618 | A | C | 0.078 | -1.18 | 0.24 |
| k__Bacteria; p__Bacteroidetes; c__Bacteroidia; o__Bacteroidales; f__Bacteroidaceae; g__Bacteroides; s__ | 7 | rs6466898 | T | C | 0.31 | -0.41 | 0.09 |
| k__Bacteria.p__Firmicutes.c__Clostridia.o__Clostridiales.f__Lachnospiraceae.g__Anaerostipes | 7 | rs10233359 | A | G | 0.075 | -0.21 | 0.04 |
| k__Bacteria; p__Firmicutes; c__Clostridia; o__Clostridiales; f__Lachnospiraceae; g__; s__ | 7 | rs10252758 | A | G | 0.493 | 1.59 | 0.33 |
| k__Bacteria; p__Firmicutes; c__Clostridia; o__Clostridiales; f__Lachnospiraceae; g__; s__ | 7 | rs10226151 | A | G | 0.48 | -1.14 | 0.24 |
| k__Bacteria.p__Firmicutes.c__Clostridia.o__Clostridiales.f__Clostridiaceae | 7 | rs6461075 | A | G | 0.288 | -0.11 | 0.02 |
| k__Bacteria; p__Firmicutes; c__Clostridia; o__Clostridiales; f__Ruminococcaceae; g__; s__ | 7 | rs7804316 | A | G | 0.325 | -2.62 | 0.57 |
| k__Bacteria; p__Firmicutes; c__Clostridia; o__Clostridiales; f__Ruminococcaceae; g__; s__ | 7 | rs10242192 | A | T | 0.07 | 3.16 | 0.61 |
| k__Bacteria; p__Firmicutes; c__Clostridia; o__Clostridiales; f__Lachnospiraceae; g__Blautia; s__ | 7 | rs17862118 | C | T | 0.139 | -3.00 | 0.58 |
| k__Bacteria; p__Bacteroidetes; c__Bacteroidia; o__Bacteroidales; f__Bacteroidaceae; g__Bacteroides | 7 | rs10488566 | G | T | 0.069 | 6.49 | 1.50 |
| k__Bacteria; p__Firmicutes; c__Clostridia; o__Clostridiales; f__Lachnospiraceae | 7 | rs10264784 | C | T | 0.12 | -0.31 | 0.07 |
| k__Bacteria_p__Firmicutes_c__Clostridia_o__Clostridiales_f__Veillonellaceae_g__Megamonas | 7 | rs7782745 | T |  | 0.05 | -0.08 | 0.19 |
| k__Bacteria.p__Lentisphaerae | 7 | rs74997447 | T |  | 0.06 | 0.00 | 0.23 |
| k__Bacteria_p__Bacteroidetes_c__Bacteroidia_o__Bacteroidales_f__.Odoribacteraceae | 7 | chr7:96414393:D | G |  | 0.06 | 0.05 | 0.31 |
| k__Bacteria.p__Firmicutes.c__Clostridia.o__Clostridiales.f__Veillonellaceae.g__Acidaminococcus | 7 | rs17739409 | C |  | 0.08 | 0.52 | 0.23 |
| k__Bacteria; p__Actinobacteria; c__Coriobacteriia; o__Coriobacteriales; f__Coriobacteriaceae; g__Eggerthella; s__lenta | 8 | rs3758054 | G | A | 0.296 | -11.00 | 2.31 |
| k__Bacteria.p__Proteobacteria.c__Gammaproteobacteria.o__Pasteurellales.f__Pasteurellaceae.g__Haemophilus | 8 | rs3808393 | T | C | 0.23 | 1.10 | 0.24 |
| k__Bacteria.p__Proteobacteria.c__Gammaproteobacteria.o__Pasteurellales.f__Pasteurellaceae.g__Haemophilus | 8 | rs1443930 | T | G | 0.365 | -0.98 | 0.21 |
| k__Bacteria; p__Firmicutes; c__Clostridia; o__Clostridiales; f__Ruminococcaceae; g__; s__ | 8 | rs1479056 | A | G | 0.409 | -1.04 | 0.23 |
| k__Bacteria; p__Firmicutes; c__Clostridia; o__Clostridiales; f__; g__; s__ | 8 | rs6999713 | C | T | 0.417 | 7.98 | 1.63 |
| k__Bacteria_p__Proteobacteria_c__Deltaproteobacteria_o__Desulfovibrionales_f__Desulfovibrionaceae_g__Desulfovibrio | 8 | rs13249293 | C |  | 0.06 | 0.01 | 0.19 |
| k__Bacteria_p__Firmicutes_c__Clostridia_o__Clostridiales_f__Veillonellaceae_g__Megamonas | 8 | rs10112815 | G |  | 0.09 | 0.04 | 0.15 |
| k__Bacteria_p__Proteobacteria_c__Deltaproteobacteria | 8 | chr8:39933549:I | CAT |  | 0.06 | 0.32 | 0.27 |
| k__Bacteria.p__Firmicutes.c__Clostridia.o__Clostridiales.f__Veillonellaceae.g__Acidaminococcus* | 8 | rs1450758 | G |  | 0.19 | 0.34 | 0.14 |
| k__Bacteria; p__Firmicutes; c__Clostridia; o__Clostridiales; f__Lachnospiraceae | 9 | rs11792081 | A | C | 0.393 | 0.79 | 0.15 |
| k__Bacteria; p__Firmicutes; c__Clostridia; o__Clostridiales; f__Ruminococcaceae; g__; s__ | 9 | rs4877854 | T | C | 0.139 | -0.98 | 0.20 |
| k__Bacteria; p__Firmicutes; c__Clostridia; o__Clostridiales; f__Lachnospiraceae; g__; s__ | 9 | rs10811270 | T | C | 0.364 | -0.95 | 0.20 |
| k__Bacteria; p__Firmicutes; c__Clostridia; o__Clostridiales; f__; g__; s__ | 9 | rs2170226 | T | C | 0.175 | -15.50 | 3.44 |
| k__Bacteria; p__Firmicutes; c__Clostridia; o__Clostridiales; f__; g__; s__ | 9 | rs586749 | A | G | 0.343 | -0.19 | 0.04 |
| k__Bacteria; p__Firmicutes; c__Clostridia; o__Clostridiales; f__Lachnospiraceae; g__; s__ | 9 | rs12552904 | A | G | 0.424 | 0.88 | 0.19 |
| k__Bacteria; p__Bacteroidetes; c__Bacteroidia; o__Bacteroidales; f__Bacteroidaceae; g__Bacteroides; s__ | 9 | rs508259 | C | T | 0.095 | -1.27 | 0.23 |
| k__Bacteria; p__Bacteroidetes; c__Bacteroidia; o__Bacteroidales; f__Bacteroidaceae; g__Bacteroides | 9 | rs10114968 | A | T | 0.064 | 7.34 | 1.52 |
| k__Bacteria_p__Firmicutes_c__Clostridia_o__SHA.98 | 9 | rs10818073 | T |  | 0.05 | -0.13 | 0.20 |
| k__Bacteria.p__Firmicutes.c__Erysipelotrichi.o__Erysipelotrichales.f__Erysipelotrichaceae.g__Clostridium | 9 | rs112294212 | T |  | 0.05 | 0.09 | 0.20 |
| k__Bacteria; p__Firmicutes; c__Clostridia; o__Clostridiales; f__Lachnospiraceae | 10 | rs7095285 | G | A | 0.463 | -0.20 | 0.05 |
| k__Bacteria; p__Firmicutes; c__Clostridia; o__Clostridiales; f__; g__; s__ | 10 | rs2505338 | T | C | 0.382 | 0.22 | 0.04 |
| k__Bacteria.p__Firmicutes.c__Clostridia.o__Clostridiales.f__Ruminococcaceae | 10 | rs1148245 | T | C | 0.351 | 0.03 | 0.01 |
| k__Bacteria; p__Firmicutes; c__Clostridia; o__Clostridiales; f__Lachnospiraceae; g__Coprococcus; s__ | 10 | rs2475321 | T | G | 0.493 | -0.20 | 0.04 |
| k__Bacteria; p__Bacteroidetes; c__Bacteroidia; o__Bacteroidales; f__Bacteroidaceae; g__Bacteroides | 10 | rs984668 | A | G | 0.368 | 8.43 | 1.62 |
| k__Bacteria; p__Firmicutes; c__Clostridia; o__Clostridiales; f__Lachnospiraceae | 10 | rs10160195 | A | G | 0.49 | 0.23 | 0.05 |
| k__Bacteria; p__Firmicutes; c__Clostridia; o__Clostridiales; f__Ruminococcaceae | 10 | rs12359026 | T | G | 0.365 | 2.07 | 0.45 |
| k__Bacteria; p__Bacteroidetes; c__Bacteroidia; o__Bacteroidales; f__Bacteroidaceae; g__Bacteroides; s__ | 10 | rs4747490 | T | G | 0.497 | -0.37 | 0.08 |
| k__Bacteria; p__Firmicutes; c__Clostridia; o__Clostridiales; f__Ruminococcaceae; g__Faecalibacterium; s__prausnitzii | 10 | rs12776647 | C | T | 0.492 | 0.09 | 0.02 |
| k__Bacteria; p__Firmicutes; c__Bacilli; o__Lactobacillales; f__Streptococcaceae; g__Streptococcus; s__ | 10 | rs2209683 | C | T | 0.228 | 5.26 | 1.02 |
| k__Bacteria; p__Bacteroidetes; c__Bacteroidia; o__Bacteroidales; f__Bacteroidaceae; g__Bacteroides; s__ | 10 | rs2997983 | C | T | 0.116 | 6.04 | 1.22 |
| k__Bacteria; p__Firmicutes; c__Clostridia; o__Clostridiales; f__Ruminococcaceae; g__Faecalibacterium; s__prausnitzii | 10 | rs2763309 | C | T | 0.242 | 0.06 | 0.01 |
| k__Bacteria; p__Firmicutes; c__Clostridia; o__Clostridiales; f__Ruminococcaceae; g__; s__ | 10 | rs10786820 | C | T | 0.276 | 0.75 | 0.16 |
| k__Bacteria; p__Firmicutes; c__Clostridia; o__Clostridiales; f__Lachnospiraceae | 10 | rs12245886 | G | T | 0.312 | 0.21 | 0.05 |
| k__Bacteria_p__Cyanobacteria_c__4C0d.2_o__YS2 | 10 | rs9421681 | T |  | 0.44 | -0.21 | 0.10 |
| k__Bacteria.p__Firmicutes.c__Bacilli.o__Lactobacillales.f__Leuconostocaceae.g__Leuconostoc | 10 | rs11188353 | T |  | 0.21 | 0.12 | 0.11 |
| k__Bacteria.p__Firmicutes.c__Clostridia.o__Clostridiales.f__Veillonellaceae.g__Acidaminococcus* | 10 | rs7899016 | T |  | 0.05 | 0.66 | 0.25 |
| k__Bacteria; p__Firmicutes; c__Clostridia; o__Clostridiales; f__Lachnospiraceae; g__; s__ | 11 | rs12577205 | C | A | 0.077 | -0.53 | 0.11 |
| k__Bacteria; p__Bacteroidetes; c__Bacteroidia; o__Bacteroidales; f__Bacteroidaceae; g__Bacteroides | 11 | rs7106602 | T | A | 0.09 | -5.98 | 1.31 |
| k__Bacteria; p__Firmicutes; c__Clostridia; o__Clostridiales; f__Lachnospiraceae; g__Blautia; s__ | 11 | rs6591450 | G | C | 0.321 | -0.20 | 0.04 |
| k__Bacteria; p__Firmicutes; c__Clostridia; o__Clostridiales; f__Ruminococcaceae; g__; s__ | 11 | rs11018543 | T | C | 0.071 | -0.43 | 0.09 |
| k__Bacteria.p__Actinobacteria.c__Actinobacteria.o__Bifidobacteriales.f__Bifidobacteriaceae | 11 | rs1367534 | T | C | 0.065 | 0.27 | 0.05 |
| k__Bacteria; p__Bacteroidetes; c__Bacteroidia; o__Bacteroidales; f__Bacteroidaceae; g__Bacteroides; s__ | 11 | rs151216 | T | C | 0.086 | -3.47 | 0.71 |
| k__Bacteria; p__Bacteroidetes; c__Bacteroidia; o__Bacteroidales; f__Bacteroidaceae; g__Bacteroides | 11 | rs7644 | T | C | 0.362 | -3.68 | 0.78 |
| k__Bacteria; p__Firmicutes; c__Clostridia; o__Clostridiales; f__Lachnospiraceae; g__; s__ | 11 | rs1193851 | G | C | 0.338 | 0.27 | 0.06 |
| k__Bacteria; p__Firmicutes; c__Clostridia; o__Clostridiales; f__; g__; s__ | 11 | rs2248020 | A | C | 0.218 | -14.10 | 3.21 |
| k__Bacteria; p__Firmicutes; c__Clostridia; o__Clostridiales; f__Lachnospiraceae; g__; s__ | 11 | rs498018 | T | G | 0.39 | 3.38 | 0.61 |
| k__Bacteria; p__Firmicutes; c__Clostridia; o__Clostridiales; f__Ruminococcaceae; g__; s__ | 11 | rs16907036 | T | G | 0.056 | -0.52 | 0.10 |
| k__Bacteria; p__Firmicutes; c__Clostridia; o__Clostridiales; f__Ruminococcaceae; g__; s__ | 11 | rs502656 | T | G | 0.177 | 0.35 | 0.07 |
| k__Bacteria; p__Firmicutes; c__Clostridia; o__Clostridiales; f__Lachnospiraceae | 11 | rs1945216 | A | G | 0.068 | 0.46 | 0.09 |
| k__Bacteria; p__Firmicutes; c__Clostridia; o__Clostridiales; f__; g__; s__ | 11 | rs641527 | A | G | 0.433 | 0.12 | 0.03 |
| k__Bacteria.p__Proteobacteria.c__Gammaproteobacteria.o__Pasteurellales.f__Pasteurellaceae.g__Haemophilus | 11 | rs10898950 | A | G | 0.224 | 1.10 | 0.24 |
| k__Bacteria; p__Firmicutes; c__Clostridia; o__Clostridiales; f__Ruminococcaceae; g__; s__ | 11 | rs2282637 | A | G | 0.061 | -4.89 | 1.10 |
| k__Bacteria; p__Bacteroidetes; c__Bacteroidia; o__Bacteroidales; f__Bacteroidaceae; g__Bacteroides; s__ | 11 | rs2282644 | C | T | 0.267 | 3.38 | 0.64 |
| k__Bacteria; p__Bacteroidetes; c__Bacteroidia; o__Bacteroidales; f__Bacteroidaceae; g__Bacteroides; s__ | 11 | rs10501839 | C | T | 0.338 | 1.89 | 0.39 |
| k__Bacteria; p__Firmicutes; c__Clostridia; o__Clostridiales; f__Ruminococcaceae; g__; s__ | 11 | rs3740874 | C | T | 0.062 | -2.08 | 0.45 |
| k__Bacteria; p__Firmicutes; c__Clostridia; o__Clostridiales; f__Lachnospiraceae; g__; s__ | 11 | rs1231637 | G | T | 0.423 | 0.26 | 0.06 |
| k__Bacteria_p__Firmicutes_c__Bacilli_o__Lactobacillales_f__Streptococcaceae_g__Lactococcus | 11 | rs11236806 | G |  | 0.22 | -0.08 | 0.15 |
| k__Bacteria; p__Bacteroidetes; c__Bacteroidia; o__Bacteroidales; f__Bacteroidaceae; g__Bacteroides | 12 | rs7296338 | G | A | 0.064 | -16.00 | 3.10 |
| k__Bacteria; p__Bacteroidetes; c__Bacteroidia; o__Bacteroidales; f__Bacteroidaceae; g__Bacteroides; s__ | 12 | rs3950612 | G | A | 0.114 | -0.33 | 0.07 |
| k__Bacteria.p__Actinobacteria.c__Coriobacteriia.o__Coriobacteriales.f__Coriobacteriaceae.g__Eggerthella | 12 | rs10879524 | G | A | 0.464 | 7.43 | 1.61 |
| k__Bacteria.p__Firmicutes.c__Clostridia.o__Clostridiales.f__Clostridiaceae | 12 | rs1234013 | C | A | 0.08 | -0.18 | 0.04 |
| k__Bacteria; p__Firmicutes; c__Clostridia; o__Clostridiales; f__Ruminococcaceae; g__; s__ | 12 | rs11831423 | A | C | 0.126 | 0.40 | 0.07 |
| k__Bacteria; p__Firmicutes; c__Clostridia; o__Clostridiales; f__Lachnospiraceae; g__; s__ | 12 | rs7979513 | A | C | 0.176 | 3.71 | 0.69 |
| k__Bacteria.p__Tenericutes.c__RF3.o__ML615J.28.f__.g__ | 12 | rs12311520 | A | C | 0.173 | -5.81 | 1.08 |
| k__Bacteria; p__Bacteroidetes; c__Bacteroidia; o__Bacteroidales; f__Bacteroidaceae; g__Bacteroides; s__ | 12 | rs1918707 | T | C | 0.447 | 34.40 | 6.95 |
| k__Bacteria; p__Firmicutes; c__Clostridia; o__Clostridiales; f__Ruminococcaceae; g__Faecalibacterium; s__prausnitzii | 12 | rs7486170 | A | G | 0.124 | 0.94 | 0.17 |
| k__Bacteria; p__Firmicutes; c__Clostridia; o__Clostridiales; f__; g__; s__ | 12 | rs7302174 | C | G | 0.206 | 0.16 | 0.03 |
| k__Bacteria; p__Firmicutes; c__Clostridia; o__Clostridiales; f__Lachnospiraceae; g__; s__ | 12 | rs1514537 | A | G | 0.255 | -0.43 | 0.09 |
| k__Bacteria; p__Firmicutes; c__Clostridia; o__Clostridiales; f__Lachnospiraceae; g__; s__ | 12 | rs10847601 | G | T | 0.232 | -2.36 | 0.44 |
| k__Bacteria; p__Firmicutes; c__Clostridia; o__Clostridiales; f__Lachnospiraceae; g__; s__ | 12 | rs7134690 | A | T | 0.225 | -1.51 | 0.29 |
| k__Bacteria.p__Firmicutes.c__Bacilli.o__Lactobacillales.f__Leuconostocaceae.g__Weissella | 12 | chr12:13622839:D | G |  | 0.10 | -0.55 | 0.15 |
| k__Bacteria_p__Firmicutes_c__Clostridia_o__Clostridiales_f__Veillonellaceae_g__Megamonas | 12 | rs11608507 | T |  | 0.22 | -0.02 | 0.11 |
| k__Bacteria_p__Firmicutes_c__Clostridia_o__Clostridiales_f__Veillonellaceae_g__Megamonas | 12 | rs17806643 | G |  | 0.15 | 0.01 | 0.12 |
| k__Bacteria; p__Firmicutes; c__Clostridia; o__Clostridiales; f__Lachnospiraceae; g__Blautia; s__ | 13 | rs1986785 | C | A | 0.496 | -2.27 | 0.43 |
| k__Bacteria.p__Tenericutes.c__Mollicutes.o__RF39 | 13 | rs4883972 | G | C | 0.438 | -0.53 | 0.10 |
| k__Bacteria; p__Bacteroidetes; c__Bacteroidia; o__Bacteroidales; f__Bacteroidaceae; g__Bacteroides; s__ | 13 | rs10507725 | A | C | 0.089 | 43.20 | 7.88 |
| k__Bacteria; p__Firmicutes; c__Clostridia; o__Clostridiales; f__Lachnospiraceae | 13 | rs9567705 | T | C | 0.08 | -0.39 | 0.09 |
| k__Bacteria; p__Firmicutes; c__Clostridia; o__Clostridiales; f__[Mogibacteriaceae]; g__; s__ | 13 | rs9548456 | T | G | 0.211 | -0.74 | 0.16 |
| k__Bacteria; p__Bacteroidetes; c__Bacteroidia; o__Bacteroidales; f__Bacteroidaceae; g__Bacteroides; s__ | 13 | rs7139612 | A | G | 0.456 | 0.37 | 0.08 |
| k__Bacteria; p__Firmicutes; c__Clostridia; o__Clostridiales; f__Ruminococcaceae; g__; s__ | 13 | rs9599240 | G | T | 0.1 | -1.87 | 0.37 |
| k__Bacteria; p__Firmicutes; c__Erysipelotrichi; o__Erysipelotrichales; f__Erysipelotrichaceae; g__Holdemania; s__ | 13 | rs9552886 | C | T | 0.151 | -0.14 | 0.03 |
| k__Bacteria; p__Firmicutes; c__Clostridia; o__Clostridiales; f__Ruminococcaceae; g__; s__ | 13 | rs6561627 | C | T | 0.074 | -2.43 | 0.50 |
| k__Bacteria; p__Bacteroidetes; c__Bacteroidia; o__Bacteroidales; f__Bacteroidaceae; g__Bacteroides; s__ | 13 | rs4360824 | C | T | 0.395 | 3.66 | 0.76 |
| k__Bacteria; p__Firmicutes; c__Clostridia; o__Clostridiales; f__; g__; s__ | 13 | rs7318523 | C | T | 0.062 | -5.38 | 1.12 |
| k__Bacteria.p__Lentisphaerae | 13 | rs9541094 | G |  | 0.08 | -0.39 | 0.18 |
| k__Bacteria.p__Actinobacteria.c__Coriobacteriia.o__Coriobacteriales.f__Coriobacteriaceae.g__Atopobium* | 13 | rs9558805 | G |  | 0.07 | -0.35 | 0.17 |
| k__Bacteria.p__Actinobacteria.c__Coriobacteriia.o__Coriobacteriales.f__Coriobacteriaceae.g__Slackia | 13 | rs36082470 | G |  | 0.17 | -0.02 | 0.13 |
| k__Bacteria; p__Firmicutes; c__Clostridia; o__Clostridiales; f__Ruminococcaceae; g__Ruminococcus | 14 | rs11845652 | G | A | 0.369 | -2.11 | 0.39 |
| k__Bacteria; p__Firmicutes; c__Clostridia; o__Clostridiales; f__; g__; s__ | 14 | rs2273623 | G | A | 0.324 | 3.77 | 0.71 |
| k__Bacteria; p__Firmicutes; c__Clostridia; o__Clostridiales; f__Lachnospiraceae; g__[Ruminococcus]; s__ | 14 | rs7156837 | G | A | 0.467 | 4.20 | 0.81 |
| k__Bacteria; p__Firmicutes; c__Clostridia; o__Clostridiales; f__Ruminococcaceae; g__; s__ | 14 | rs922408 | G | A | 0.289 | -2.74 | 0.59 |
| k__Bacteria; p__Firmicutes; c__Clostridia; o__Clostridiales; f__Lachnospiraceae; g__Coprococcus; s__ | 14 | rs2293702 | T | C | 0.477 | 0.08 | 0.01 |
| k__Bacteria; p__Firmicutes; c__Clostridia; o__Clostridiales; f__; g__; s__ | 14 | rs10148020 | G | C | 0.065 | 3.06 | 0.55 |
| k__Bacteria; p__Firmicutes; c__Clostridia; o__Clostridiales; f__Lachnospiraceae; g__Blautia; s__ | 14 | rs17585587 | G | C | 0.164 | -1.60 | 0.33 |
| k__Bacteria; p__Bacteroidetes; c__Bacteroidia; o__Bacteroidales; f__Bacteroidaceae; g__Bacteroides; s__ | 14 | rs4901725 | C | T | 0.355 | -0.32 | 0.06 |
| k__Bacteria; p__Firmicutes; c__Clostridia; o__Clostridiales; f__Ruminococcaceae; g__; s__ | 14 | rs1128880 | G | T | 0.486 | -2.67 | 0.52 |
| k__Bacteria; p__Firmicutes; c__Clostridia; o__Clostridiales; f__Ruminococcaceae; g__; s__ | 14 | rs11621258 | A | T | 0.319 | 1.06 | 0.24 |
| k__Bacteria; p__Firmicutes; c__Clostridia; o__Clostridiales; f__; g__; s__ | 14 | rs1278911 | G | T | 0.348 | 12.20 | 2.77 |
| k__Bacteria; p__Firmicutes; c__Clostridia; o__Clostridiales; f__Lachnospiraceae; g__; s__ | 14 | rs718362 | C | T | 0.14 | 0.35 | 0.08 |
| k__Bacteria_p__Firmicutes_c__Bacilli_o__Lactobacillales_f__Streptococcaceae_g__Lactococcus | 14 | rs10148302 | G |  | 0.22 | -0.08 | 0.14 |
| k__Bacteria; p__Firmicutes; c__Clostridia; o__Clostridiales; f__[Mogibacteriaceae]; g__; s__ | 15 | rs12900413 | G | A | 0.28 | 0.67 | 0.14 |
| k__Bacteria; p__Bacteroidetes; c__Bacteroidia; o__Bacteroidales; f__Bacteroidaceae; g__Bacteroides; s__ | 15 | rs4777927 | T | C | 0.336 | 0.24 | 0.04 |
| k__Bacteria; p__Firmicutes; c__Clostridia; o__Clostridiales; f__Veillonellaceae; g__Veillonella; s__dispar | 15 | rs347941 | A | C | 0.231 | 4.76 | 0.92 |
| k__Bacteria; p__Firmicutes; c__Clostridia; o__Clostridiales; f__Ruminococcaceae; g__; s__ | 15 | rs16968570 | G | C | 0.077 | -1.83 | 0.41 |
| k__Bacteria; p__Firmicutes; c__Clostridia; o__Clostridiales; f__Ruminococcaceae; g__; s__ | 15 | rs730589 | T | G | 0.337 | -1.20 | 0.21 |
| k__Bacteria; p__Firmicutes; c__Clostridia; o__Clostridiales; f__; g__; s__ | 15 | rs2007084 | A | G | 0.091 | -0.54 | 0.10 |
| k__Bacteria; p__Firmicutes; c__Clostridia; o__Clostridiales; f__Ruminococcaceae; g__; s__ | 15 | rs16977800 | A | G | 0.103 | 5.31 | 1.04 |
| k__Bacteria; p__Firmicutes; c__Clostridia; o__Clostridiales; f__Ruminococcaceae; g__Faecalibacterium; s__prausnitzii | 15 | rs12915891 | T | G | 0.225 | 0.64 | 0.13 |
| k__Bacteria; p__Firmicutes; c__Clostridia; o__Clostridiales; f__Lachnospiraceae; g__; s__ | 15 | rs1874835 | T | G | 0.116 | -0.60 | 0.13 |
| k__Bacteria; p__Bacteroidetes; c__Bacteroidia; o__Bacteroidales; f__Bacteroidaceae; g__Bacteroides | 15 | rs16948999 | C | T | 0.202 | 4.20 | 0.92 |
| k__Bacteria; p__Bacteroidetes; c__Bacteroidia; o__Bacteroidales; f__Bacteroidaceae; g__Bacteroides | 15 | rs11073804 | A | T | 0.182 | -4.47 | 0.99 |
| k__Bacteria; p__Firmicutes; c__Clostridia; o__Clostridiales; f__; g__; s__ | 16 | rs9938742 | G | A | 0.47 | -11.90 | 2.69 |
| k__Bacteria.p__Actinobacteria.c__Coriobacteriia.o__Coriobacteriales.f__Coriobacteriaceae.g__Eggerthella | 16 | rs17299191 | T | C | 0.241 | 8.91 | 1.81 |
| k__Bacteria; p__Firmicutes; c__Clostridia; o__Clostridiales; f__; g__; s__ | 16 | rs7499858 | A | G | 0.136 | -4.97 | 0.92 |
| k__Bacteria; p__Firmicutes; c__Clostridia; o__Clostridiales; f__Lachnospiraceae; g__Coprococcus; s__ | 16 | rs722075 | A | G | 0.181 | 0.32 | 0.07 |
| k__Bacteria; p__Actinobacteria; c__Coriobacteriia; o__Coriobacteriales; f__Coriobacteriaceae; g__Eggerthella; s__lenta | 16 | rs1420227 | C | T | 0.253 | 12.20 | 2.38 |
| k__Bacteria; p__Firmicutes; c__Clostridia; o__Clostridiales; f__Lachnospiraceae | 16 | rs1955390 | C | T | 0.345 | -0.21 | 0.05 |
| k__Bacteria; p__Bacteroidetes; c__Bacteroidia; o__Bacteroidales; f__Bacteroidaceae; g__Bacteroides; s__ | 17 | rs11868220 | G | A | 0.365 | -2.85 | 0.61 |
| k__Bacteria; p__Bacteroidetes; c__Bacteroidia; o__Bacteroidales; f__Bacteroidaceae; g__Bacteroides | 17 | rs3105827 | T | A | 0.092 | 5.84 | 1.30 |
| k__Bacteria.p__Firmicutes.c__Clostridia.o__Clostridiales.f__Clostridiaceae | 17 | rs228307 | C | A | 0.079 | -0.18 | 0.04 |
| k__Bacteria; p__Firmicutes; c__Clostridia; o__Clostridiales; f__Ruminococcaceae; g__; s__ | 17 | rs9889895 | A | G | 0.119 | 2.14 | 0.41 |
| k__Bacteria; p__Bacteroidetes; c__Bacteroidia; o__Bacteroidales; f__Bacteroidaceae; g__Bacteroides; s__ | 17 | rs12936861 | T | G | 0.346 | -37.50 | 7.36 |
| k__Bacteria; p__Firmicutes; c__Clostridia; o__Clostridiales; f__; g__; s__ | 17 | rs17763551 | A | G | 0.175 | 3.57 | 0.70 |
| k__Bacteria; p__Firmicutes; c__Clostridia; o__Clostridiales; f__; g__; s__ | 17 | rs4968435 | A | G | 0.084 | 13.40 | 2.89 |
| k__Bacteria; p__Firmicutes; c__Clostridia; o__Clostridiales; f__Ruminococcaceae; g__; s__ | 17 | rs12951596 | A | G | 0.134 | 1.52 | 0.33 |
| k__Bacteria_p__Firmicutes_c__Bacilli_o__Lactobacillales_f__Lactobacillaceae_g__Lactobacillus | 17 | rs76906435 | C |  | 0.08 | 0.02 | 0.16 |
| k__Bacteria; p__Firmicutes; c__Clostridia; o__Clostridiales; f__Lachnospiraceae; g__Dorea; s__ | 18 | rs12607607 | T | C | 0.169 | -0.63 | 0.11 |
| k__Bacteria; p__Firmicutes; c__Clostridia; o__Clostridiales; f__Ruminococcaceae; g__; s__ | 18 | rs495078 | T | C | 0.387 | -2.90 | 0.55 |
| k__Bacteria; p__Firmicutes; c__Clostridia; o__Clostridiales; f__Lachnospiraceae; g__Coprococcus; s__ | 18 | rs12606140 | T | C | 0.169 | -0.35 | 0.07 |
| k__Bacteria; p__Firmicutes; c__Clostridia; o__Clostridiales; f__Lachnospiraceae; g__; s__ | 18 | rs1922100 | T | C | 0.201 | 1.16 | 0.24 |
| k__Bacteria; p__Firmicutes; c__Clostridia; o__Clostridiales; f__Lachnospiraceae; g__; s__ | 18 | rs11082729 | T | C | 0.153 | -1.25 | 0.27 |
| k__Bacteria.p__Firmicutes.c__Clostridia.o__Clostridiales.f__Lachnospiraceae.g__Roseburia | 18 | rs7231241 | A | G | 0.075 | 0.18 | 0.04 |
| k__Bacteria; p__Firmicutes; c__Clostridia; o__Clostridiales; f__Ruminococcaceae; g__Faecalibacterium; s__prausnitzii | 18 | rs17079032 | C | T | 0.156 | -0.21 | 0.04 |
| k__Bacteria; p__Verrucomicrobia; c__Verrucomicrobiae; o__Verrucomicrobiales; f__Verrucomicrobiaceae; g__Akkermansia; s__muciniphila | 18 | rs692899 | C | T | 0.385 | -0.55 | 0.11 |
| k__Bacteria; p__Firmicutes; c__Clostridia; o__Clostridiales; f__; g__; s__ | 18 | rs7236263 | C | T | 0.363 | 7.89 | 1.67 |
| k__Bacteria; p__Bacteroidetes; c__Bacteroidia; o__Bacteroidales; f__Bacteroidaceae; g__Bacteroides; s__ | 18 | rs470497 | C | T | 0.405 | 0.57 | 0.12 |
| k__Bacteria_p__Proteobacteria_c__Deltaproteobacteria_o__Desulfovibrionales_f__Desulfovibrionaceae_g__Desulfovibrio | 18 | rs12457158 | C |  | 0.16 | 0.18 | 0.13 |
| k__Bacteria; p__Firmicutes; c__Clostridia; o__Clostridiales; f__Lachnospiraceae; g__; s__ | 19 | rs11880147 | G | A | 0.197 | 5.21 | 0.93 |
| bray_curtis_PC3 | 19 | rs3859542 | T | C | 0.174 | -0.03 | 0.00 |
| k__Bacteria; p__Bacteroidetes; c__Bacteroidia; o__Bacteroidales; f__[Barnesiellaceae]; g__; s__ | 19 | rs4897946 | A | G | 0.226 | 19.00 | 3.62 |
| k__Bacteria; p__Firmicutes; c__Clostridia; o__Clostridiales; f__Ruminococcaceae; g__; s__ | 19 | rs350916 | A | G | 0.49 | -2.73 | 0.57 |
| k__Bacteria; p__Firmicutes; c__Clostridia; o__Clostridiales; f__Ruminococcaceae; g__; s__ | 19 | rs17242235 | A | G | 0.149 | 1.47 | 0.31 |
| k__Bacteria; p__Bacteroidetes; c__Bacteroidia; o__Bacteroidales; f__Bacteroidaceae; g__Bacteroides | 19 | rs7258176 | C | T | 0.371 | -3.49 | 0.79 |
| k__Bacteria_p__Bacteroidetes_c__Bacteroidia_o__Bacteroidales_f__.Odoribacteraceae | 19 | rs115795847 | T |  | 0.06 | 0.54 | 0.27 |
| k__Bacteria; p__Firmicutes; c__Clostridia; o__Clostridiales; f__Ruminococcaceae; g__Faecalibacterium; s__prausnitzii | 20 | rs1320561 | G | A | 0.426 | -0.55 | 0.11 |
| k__Bacteria; p__Firmicutes; c__Clostridia; o__Clostridiales; f__Lachnospiraceae; g__; s__ | 20 | rs404775 | G | A | 0.484 | 0.38 | 0.08 |
| k__Bacteria; p__Bacteroidetes; c__Bacteroidia; o__Bacteroidales; f__Bacteroidaceae; g__Bacteroides; s__ | 21 | rs11701867 | A | C | 0.174 | 0.59 | 0.11 |
| k__Bacteria.p__Firmicutes.c__Clostridia.o__Clostridiales.f__Lachnospiraceae.g__Roseburia | 21 | rs2832648 | A | G | 0.125 | 0.16 | 0.03 |
| k__Bacteria; p__Firmicutes; c__Clostridia; o__Clostridiales; f__; g__; s__ | 21 | rs2830259 | C | T | 0.194 | 14.60 | 3.37 |
| k__Bacteria_p__Firmicutes_c__Clostridia_o__Clostridiales_f__Lachnospiraceae_g__Pseudobutyrivibrio | 21 | rs35197943 | T |  | 0.13 | 0.13 | 0.15 |
| k__Bacteria.p__Proteobacteria.c__Gammaproteobacteria.o__Pasteurellales.f__Pasteurellaceae.g__Haemophilus | 22 | rs6007059 | G | A | 0.146 | -1.31 | 0.29 |
| k__Bacteria; p__Firmicutes; c__Clostridia; o__Clostridiales; f__; g__; s__ | 22 | rs5754822 | C | A | 0.317 | 12.10 | 2.82 |
| k__Bacteria; p__Firmicutes; c__Clostridia; o__Clostridiales; f__; g__; s__ | 22 | rs5997791 | A | C | 0.23 | 9.50 | 1.93 |
| k__Bacteria.p__Firmicutes.c__Clostridia.o__Clostridiales.f__Ruminococcaceae | 22 | rs3788704 | T | C | 0.115 | -0.03 | 0.01 |
| k__Bacteria.p__Lentisphaerae | 22 | rs1800694 | T |  | 0.05 | 0.24 | 0.27 |
